# Pelargonic Acid: From Historical Uses to Future Perspectives in Sustainable Agriculture

**DOI:** 10.1021/acs.jafc.5c14438

**Published:** 2026-03-03

**Authors:** Ana Cristina Preisler, Estefânia Vangelie Ramos Campos, Vanessa Takeshita, Jéssica S. Rodrigues, Amanda de S. M. de Freitas, Brian Cintra Cardoso, Lais D. Battaglini, Leonardo Fernandes Fraceto

**Affiliations:** † Institute of Science and Technology, 28108 Sao Paulo State University, Av. Três de Março, 511–Alto da Boa Vista, 18087-180 Sorocaba, São Paulo, Brazil; ‡ B. Nano Soluções Tecnológicas LTDA, Avenida Itavuvu, 11777 Sorocaba, São Paulo, Brazil

**Keywords:** nonanoic acid, bioherbicide, weed control, bioeconomy, nature-based solutions

## Abstract

Pelargonic acid (PA), or nonanoic acid, is a saturated fatty acid with a nine-carbon chain and low toxicity. Although initially applied in processes, PA has gained relevance in agriculture as a bioherbicide, driven by demand for sustainable alternatives to synthetic herbicides. Recently approved for agricultural use in several countries, PA has demonstrated effective control of a range of weed species; however, its agricultural use faces limitations, such as complexity of synthesis, high volatility, degradation in field, and need for frequent applications. As a contact herbicide, PA acts through rapid membrane disruption that underlies its burndown effect but requires high application rates and repeated treatments in systems. This review provides an analysis of PA, addressing its relevance and consolidation as a bioherbicide with sustainability goals, discussing transition from conventional herbicides to molecules. It highlights that nanotechnology-based approaches can address limitations, improving efficacy and persistence, supporting integration into management.

## Graphical abstract

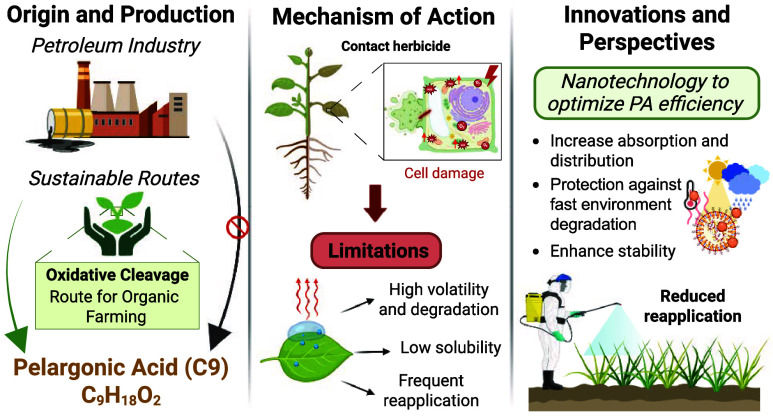

## 1 Introduction

Also known as nonanoic acid, pelargonic acid (PA) is a saturated fatty acid with a linear nine-carbon chain (C_9_H_18_O_2_). It occurs naturally in small amounts in essential oils from various plants, such as *Pelargonium* spp., and from vegetable and animal lipid sources. PA is a molecule that degrades rapidly in soil, has low solubility in water (0.28 g L^–1^ at 30 °C), a p*K*_a_ of 4.96, and a low risk of toxicity.^1,2^ The molecular structure comprises a monofunctional carboxylic acid (HOOC­(CH_2_)_7_CH_3_), which can be obtained through the oxidative cleavage of unsaturated fatty acids such as oleic acid, gondoic acid, and erucic acid. This can generate different byproducts depending on the precursor used.^3^

Initially, its applications were primarily industrial, and it was used in the formulation of plasticizers, lubricants, surfactants, and fragrances. PA has been gaining attention owing to its biological properties, particularly in agriculture.^3–7^ In 1995, Mycogen Corporation introduced PA to the pesticide market under the brand name Scythe, containing 57% w/w PA, which was its first large-scale commercial use as a natural herbicide.^4^

PA-based products, such as Beloukha, marketed by companies such as JADE, have expanded into the European market. Initial registration in France occurred in 2014, followed by approval from Italy and other EU countries.^3,6^ The formulation was designed for postemergence, nonselective contact and can be applied in areas such as streets, roadsides, ornamental landscapes, residential gardens, and community gardens. Its broad-spectrum activity includes annual and perennial monocotyledonous and dicotyledonous species,^7–10^ particularly during the early stages of growth. In Brazil, PA is registered for use in organic agriculture, provided that it is obtained from natural or renewable sources, such as through the oxidative cleavage of oleic acid.^11^ However, no commercial product is yet available. This international recognition reflects regulatory openness and the growing societal demand for natural weed-control agents.

Among the currently available broad-spectrum natural herbicides, PA is the most widely used, with commercial emulsifiable concentrate formulations registered in multiple regions and field application rates commonly corresponding to 16–32 L ha^–1^ of formulations containing around 680 g L^–1^ of active ingredient, resulting in high applied doses. First extracted from the leaves of *Pelargonium roseum*, it has been recognized as a contact herbicide with broad-spectrum activity.^9^ Unlike systemic herbicides, which are translocated through the plant’s vascular system and affect distant tissues, PA disrupts epidermal cell membranes, leading to rapid dehydration and the loss of cellular function, a well-documented contact effect at the cellular level. Its fast-acting, broad-spectrum contact action distinguishes it from other bioherbicides, making it a versatile candidate for integrated weed management.

Although herbicidal effects have been reported, their mechanism of action remains unknown. The herbicidal activity of fatty acids varies substantially depending on chain length. Medium-chain fatty acids, such as PA, exhibit strong herbicidal effects. Meanwhile, shorter-chain (C6) or longer-chain (C14) saturated fatty acids have little or no activity in weed control.^3,12,13^

Despite its advantages, such as rapid degradation, low environmental persistence, and minimal bioaccumulation risk, PA requires more frequent reapplication at shorter intervals than conventional herbicides. This is because of its rapid volatilization and short-field persistence. To improve its performance, formulations incorporating surfactants and emulsifiers have been developed to enhance solubility, wettability, and uniform adhesion to the leaf surface.^1,14–16^ Combinations with other synthetic and nonsynthetic compounds, such as acetic acid and other allelochemical compounds, have been examined to potentiate herbicidal effects and optimize weed control at different development stages or under varying environmental conditions.^2,16^

The rapid advancement of weed resistance to synthetic herbicides has become a major driver in the search for alternative weed control strategies. Currently, 269 resistant weed species and 523 cases (species × site of action) have been reported worldwide, covering 21 of the 31 mechanisms of action available on the market. In parallel, growing environmental concerns associated with synthetic herbicides, such as glyphosate, diquat, and atrazine, have increased the demand for alternative solutions that minimize soil and water contamination while mitigating resistance development in weed populations.^17,18^

In a more worrying scenario, cases of multiple resistance involving 2–11 simultaneous mechanisms of action have already been reported, indicating a loss of efficiency in chemical management as a tool for controlling weeds worldwide. Despite the adoption of diverse herbicide mechanisms of action in agricultural systems, recent strategies have proven less effective in reducing the incidence of herbicide-resistant weed species. In this context, PA is a valuable tool for effective and environmentally safe weed management, particularly in organic agriculture.^16^

However, its application still faces challenges in formulation optimization, field performance, low delivery efficiency, and cost-effectiveness relative to conventional herbicides. To address these limitations, nanotechnology is a promising approach for improving the stability, efficacy, and selectivity of bioherbicides. Nanocarrier-based formulations enable a more controlled release of PA, enhancing its chemical stability, promoting better foliar adhesion, and reducing volatilization and premature degradation. These approaches reduce the frequency of reapplication and minimize environmental impacts. Functionalized nanoparticles can also increase retention on target plant surfaces and improve absorption efficiency while minimizing unintended effects on nontarget crops.^19^

Given the increasing regulatory restrictions on synthetic herbicides and the urgent need for environmentally friendly weed management solutions, it is critical to assess the role of PA in this evolving agricultural landscape. Recognized for its natural origin and contact herbicidal activity, PA is a promising tool in this context. However, despite their potential, knowledge gaps remain regarding their optimization, long-term effectiveness, and integration with news technologies. Recent advances in nanotechnology have offered new opportunities to enhance performance. However, a full review of these developments is lacking. Therefore, this review examines pelargonic acid from its early agricultural applications to its establishment as a bioherbicide within sustainability-driven weed management approaches. The discussion addresses the shifting paradigm of weed control, including the transition from synthetic herbicides to natural alternatives and the role of nanotechnology in improving PA efficiency. By considering both the challenges and opportunities associated with its application, this review provides insight into the potential contribution of PA to sustainable weed management strategies.

## 2 Routes and Synthesis Processes for PA

Fossil fuel reserve depletion, coupled with growing environmental concerns, has intensified the search for renewable alternatives to petroleum-derived chemicals used in agricultural and agrochemical applications.^20^ Among these alternatives, oils and fats of vegetable and animal origin are promising raw materials, with economic viability, biodegradability, and functional versatility.^21,22^ Their chemical structures make them highly versatile for industrial applications because they combine long hydrocarbon chains similar to those found in petroleum-derived compounds with multiple reactive functional groups that are suitable for chemical modifications.^23^ Their natural abundance, biodegradability, and nontoxic properties make them attractive alternatives to conventional petrochemical materials.^1,24^ This also positions them as ideal feedstocks for PA synthesis, which benefits from their renewable nature and chemical adaptability.^1,16^

The oleochemical sector has grown considerably in recent years, with the global vegetable oilseed production projected to reach 677 million metric tons by 2034, yielding approximately 207 million metric tons of vegetable oil.^25^ These oils are key raw materials for surfactants, lubricants, polymers, and biobased specialty chemicals, including PA.^26^ Their broad availability and sustainability make them attractive candidates for industrial applications that aim to replace fossil-based products.

Triglycerides, the primary constituents of vegetable and animal fats, are composed of glycerol and three fatty acids, typically palmitic, oleic, and α-linolenic acids. Triglyceride composition varies by source. Animal fats contain a higher proportion of saturated fatty acids. Meanwhile, vegetable oils are richer in unsaturated fatty acids.^27^ Among these, oleic acid (C18:1) is of particular interest because it is a key precursor for PA production. Through oxidative cleavage, oleic acid is transformed into PA (C9 monocarboxylic acid) and azelaic acid (C9 dicarboxylic acid), both of which have valuable applications in agricultural, cosmetic, and polymer industries.^15^

The development of sustainable and efficient synthetic routes for PA is crucial for its large-scale commercial application. Various chemical and biotechnological approaches have been explored to optimize the oxidative cleavage of oleic acid and balance factors, such as yield, environmental impact, and industrial feasibility. The following sections provide in-depth discussions of the main synthetic pathways, their advantages and challenges, and their potential for large-scale production.

### 2.1 Comparing Natural Sources and Synthetic Pathways

PA can be naturally sourced from the essential oils of *Pelargonium* species (*Pelargonium* spp.), which are among the richest plant-based sources of this n-carbon fatty acid. The extraction process involves steam-distillation of the aerial parts of the plant, followed by liquid–liquid extraction or chromatographic techniques to purify the PA fraction. Although the concentration of PA in *Pelargonium* EOs is relatively low compared to other biotechnological sources, its natural origin aligns with the requirements for organic-certified agricultural inputs.^1,15^

PA synthesis has been extensively explored through various routes, which can be broadly categorized into petrochemical- and biobased sustainable approaches. While petrochemical processes remain dominant owing to their industrial scalability and efficiency, environmental concerns and regulatory restrictions are driving the transition toward sustainable pathways that use vegetable oils and animal fats. Among the most abundant biobased sources, oleic acid (C18:1), which is a monounsaturated fatty acid found in various vegetable oils and animal fats, is widely used as a precursor for PA production.^23^

Oxidative cleavage of olefins plays a crucial role in PA synthesis, involving the breaking of carbon–carbon double bonds and their conversion into carbon–oxygen bonds. This can be achieved via different oxidation routes, ranging from ozonolysis to metal-catalyzed oxidative scission.^28^ Ozone (O_3_), an allotrope of oxygen, reacts rapidly with carbon–carbon double bonds, enabling the conversion of alkenes into aldehydes or carboxylic acids without the need for a metal catalyst.^29,30^ Azelaic acid (C9, dicarboxylic acid) is an industrially significant compound primarily produced on a large scale through the ozonolysis of oleic acid. This process generates PA (C9, monocarboxylic acid) as a byproduct.^31^ However, despite being a byproduct, PA holds considerable value as a chemical with various industrial applications.^31,32^

Figure 1 shows the main synthetic pathways for PA production, differentiating between traditional petrochemical and sustainable biobased methods.

**Figure 1:**
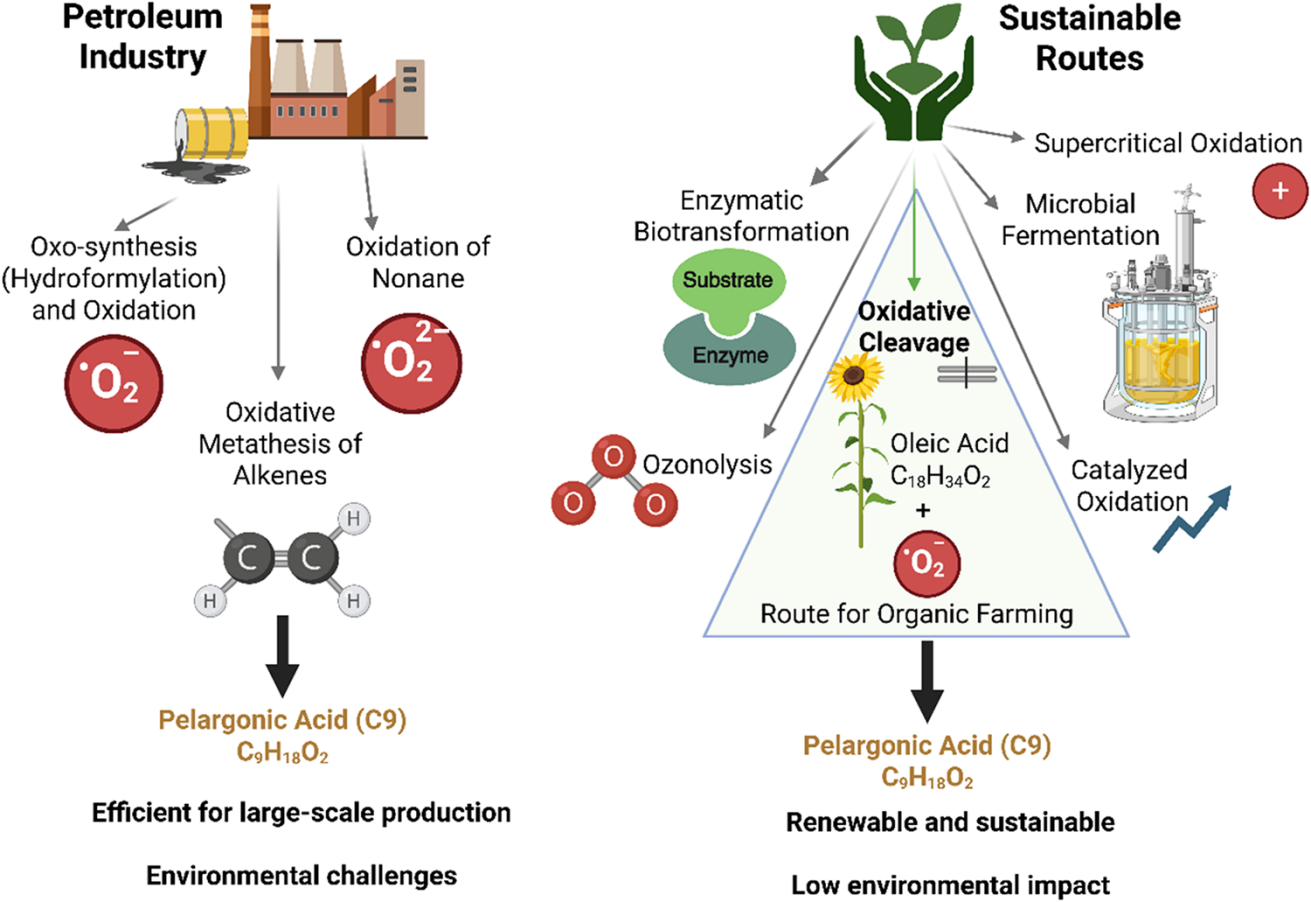
Main routes for the production of pelargonic acid (C_9_H_18_O_2_) from petroleum industry and sustainable routes. The processes include, oxo-synthesis (hydroformylation) and oxidation, oxidative metathesis of alkenes, ozonolysis, catalyzed oxidation, supercritical oxidation, microbial fermentation, and enzymatic biotransformation, resulting in the conversion of the substrate into pelargonic acid.

Table 1 summarizes the main synthetic pathways for PA production, highlighting key reactions, environmental challenges, and costs associated with each route.

**Table 1:** Comparison of the Main Synthetic Pathways for Pelargonic Acid (PA) Production, Highlighting Key Chemical Reactions, Environmental Challenges, and Associated Costs^a^

main routes	chemical description	overall conversion scheme	environmental challenges	associated costs	refs
oxidation of nonanal	selective oxidation of nonanal (C_9_ aldehyde) to pelargonic acid using oxidants or catalytic methods	${\mathrm{CH}}_{3}{\left({\mathrm{CH}}_{2}\right)}_{7}\mathrm{CHO}+\frac{1}{2}O_{2}\to C_{9}H_{18}O_{2}$	need for controlled reaction conditions to avoid overoxidation	moderate cost due to raw material availability and catalyst use	Davies et al. (2016)
oxo-synthesis (hydroformylation) and oxidation	alkenes react with CO/H_2_ forming aldehydes, which are subsequently oxidized to pelargonic acid	${\mathrm{CH}}_{3}{\left({\mathrm{CH}}_{2}\right)}_{6}\mathrm{CH}={\mathrm{CH}}_{2}+\mathrm{CO}+H_{2}\to {\mathrm{CH}}_{3}{\left({\mathrm{CH}}_{2}\right)}_{7}\mathrm{CHO}$	use of metal catalysts, high pressure, and CO emissions	hgh cost of catalysts and high-pressure infrastructure	Frohning et al. (1996)
		$${\mathrm{CH}}_{3}{\left({\mathrm{CH}}_{2}\right)}_{7}\mathrm{CHO}+\left[O\right]\to C_{9}H_{18}O_{2}$$			
oxidative metathesis of alkenes	cleavage and reorganization of alkenes using metal catalysts and oxidants	${\mathrm{CH}}_{3}{\left({\mathrm{CH}}_{2}\right)}_{7}\mathrm{CH}={\mathrm{CH}}_{2}\to C_{9}H_{18}O_{2}+\mathrm{subproducts}$	dependence on noble metals and variable efficiency	expensive catalysts and strict control of side reactions	Goldbach et al. (2015)
ozonolysis	oxidative cleavage of alkenes with ozone, forming carboxylic acids	$C_{18}H_{34}O_{2}+2O_{3}\to C_{9}H_{18}O_{2}+C_{9}H_{16}O_{4+}O_{2}$	formation of unstable ozonides requiring safe handling	high reagent consumption and specialized equipment	Atalpalkar et al. (2020)
catalyzed oxidation	direct oxidation of fatty acids or alkenes using metal or enzymatic catalysts	$C_{18}H_{34}O_{2}+4H_{2}O_{2}\overset{{\mathrm{WO}}_{3}}{\to }C_{9}H_{18}O_{2}+C_{9}H_{16}O_{4}+4H_{2}O$	potential catalyst toxicity and need for recovery	moderate cost of catalysts and recycling processes	Hood et al. (2020); Li et al. (2018)
supercritical oxidation	use of supercritical water or CO_2_ to oxidize substrates	$C_{18}H_{34}O_{2}+O_{2}\to C_{9}H_{18}O_{2}+C_{9}H_{16}O_{4}$	extreme temperature and pressure conditions, high energy consumption.	high operational cost and need for robust equipment.	Sparks et al. (2009)
oxidative cleavage	fatty acids or alkenes undergo oxidative cleavage using peroxides, oxygen, or catalytic systems	$C_{18}H_{34}O_{2}+\left[O\right]\to C_{9}H_{18}O_{2}+C_{9}H_{16}O_{4}$	use of strong oxidants, risk of byproducts	moderate to high cost depending on the oxidant and catalyst employed	Antonelli et al. (1998); Benessere et al. (2015)
microbial fermentation	conversion of fatty acids by microorganisms into pelargonic acid	$C_{n}H_{2n}O_{2}+\left[O\right]\to \left(m\mathrm{icrobial}\;e\mathrm{nzymes}\right)\to C_{9}H_{18}O_{2}+{\mathrm{CO}}_{2}+H_{2}O$	long reaction times and need for biotechnological optimization	moderate cost but requires bioprocess infrastructure	Lee et al. (2018); Makvandi et al. (2021); Li et al. (2020)
enzymatic biotransformation	selective oxidation of fatty acids by specific enzymes	$C_{18}H_{34}O_{2}+4H_{2}O_{2}\to \left(\mathrm{lipase}+\mathrm{oxidase}\right)\to C_{9}H_{18}O_{2}+C_{9}H_{16}O_{4}+4H_{2}O$	enzyme stability and yield depend on reaction conditions	high production costs and need for enzyme reuse	Mao et al. (2024); Brenna et al. (2020)

a The table contrasts petrochemical and bio-based methods, considering factors such as reagent requirements, process efficiency, and sustainability aspects.

#### 2.1.1 Petrochemical Routes for PA Production

Petrochemical methods represent the most established industrial routes for PA production, offering high yields, mature processing infrastructure, and scalability.^28,31^ Although increasing environmental and regulatory pressures favor renewable alternatives, fossil-based approaches remain widely adopted in large-scale manufacturing. Table 1 summarizes key synthetic pathways, including petrochemical and biobased methods, along with associated reaction types, limitations, and cost considerations.

A conventional petrochemical strategy involves the selective oxidation of nonanal (C_9_ aldehyde), typically using molecular oxygen or peroxides in the presence of transition metal catalysts such as cobalt or manganese salts. This reaction proceeds under mild conditions but requires careful control of temperature and oxidant concentration to avoid overoxidation and the formation of byproducts, including dicarboxylic acids (e.g., azelaic acid), short-chain cleavage products, and insoluble condensation residues. Oxo-synthesis, based on hydroformylation of alkenes followed by oxidation, has been reported for PA production, although its complexity and reliance on noble metal catalysts limit agricultural applicability (see Table 1).^33,34^

Oxidative metathesis of unsaturated fatty acid derivatives has been explored as an alternative route to oxygenated C_9_ products, such as PA and azelaic acid.^35^ This method employs ruthenium- or molybdenum-based catalysts to cleave and reorganize alkenes via alkene metathesis. While offering excellent selectivity, practical limitations include low throughput, high catalyst cost, and strict requirements for inert and controlled environments.

#### 2.1.2 Bio-Based Routes for Sustainable PA Production

The transition from petroleum-based to biobased routes for PA production has gained increasing attention in recent years, driven by sustainability demands, regulatory pressure, and the availability of renewable lipid feedstocks.^15,36,37^ Among these, oleic acid-rich vegetable oils have emerged as key raw materials, enabling the conversion of long-chain fatty acids into medium-chain fatty acids via oxidative cleavage. These biobased approaches aim to balance efficiency, safety, environmental performance, and compatibility with agricultural applications.

Ozonolysis is one of the most established oxidative cleavage methods for converting oleic acid into PA and azelaic acid.^30^ This approach offers high selectivity and product purity and has been extensively investigated at laboratory and industrial scales. However, the formation of unstable ozonide intermediates requires stringent safety protocols, controlled decomposition steps, and specialized equipment, significantly limiting scalability and economic attractiveness for agricultural applications.^30^

To overcome the safety limitations associated with ozonolysis, hydrogen peroxide-based oxidation has emerged as a promising alternative. When combined with tungsten- or molybdenum-based catalysts, hydrogen peroxide enables efficient oxidative cleavage under milder conditions while reducing operational hazards.^38^ This approach has demonstrated competitive yields of PA and azelaic acid, positioning it as a safer and more adaptable route for biobased production. Representative studies illustrate the potential of hydrogen peroxide-mediated oxidation. Antonelli et al. reported that PA yields exceed 80% with tungstate-based catalytic systems. At the same time, Li et al. demonstrated improved oxidant efficiency through catalyst stabilization strategies, achieving moderate to high yields of PA alongside azelaic acid. Despite these advances, catalyst cost, recovery, and long-term stability remain essential considerations for industrial implementation.

Supercritical oxidation has also been investigated as a high-efficiency route for oleic acid cleavage, benefiting from enhanced mass transfer and oxidant solubility under supercritical CO_2_. Conversion rates above 95% have been reported, outperforming conventional ozonolysis. However, the requirement for high-pressure reactors and complex operational controls severely limits its scalability.^39^

Despite notable progress, biobased oxidative routes still face challenges related to catalyst toxicity, process complexity, and overall sustainability. The use of strong oxidants, catalyst deactivation, and difficulties associated with catalyst recycling can compromise economic and environmental performance. Consequently, continued efforts are required to develop safer, cost-effective, and scalable oxidative cleavage technologies suitable for agricultural deployment.

Beyond chemical oxidation, microbial fermentation has emerged as a renewable alternative for PA production using nonfood biomass and mild operating conditions.^40^ These biological routes avoid harsh reagents and high-energy inputs; however, challenges related to productivity, strain robustness, and downstream processing still limit their large-scale adoption. Enzymatic and chemoenzymatic strategies provide additional opportunities for selective PA production. Enzyme-mediated transformations enable high specificity under mild conditions, while hybrid systems combining biocatalysis with controlled chemical oxidation have demonstrated improved efficiency. Notably, one-pot chemoenzymatic processes converting oleic acid into PA and azelaic acid have achieved high yields while minimizing harsh reagents and purification steps.^41–43^

Overall, biobased routes for PA production exhibit distinct advantages and limitations in terms of yield, safety, cost, and regulatory acceptance. Comparative evaluation of these approaches is essential for guiding industrial and agricultural implementation. In Brazil, for example, PA is approved for agricultural use only when derived from renewable plant-based sources or synthesized via oxidative cleavage of fatty acids, explicitly excluding petrochemical pathways.^11^

## 3 Mode of Action in Plants and Environmental Dynamics of PA

### 3.1 Contact Herbicide Activity and Phytotoxic Responses

PA is marketed globally by some companies, primarily as an alternative to synthetic herbicides such as glyphosate. However, its primary molecular mode of action and specific target site remain unclassified by the Herbicide Resistance Action Committee.^18^ As an active ingredient, PA is regarded as a contact burndown herbicide, with rapid phytotoxic effects observed.^1,44,45^ Plants and cells are rapidly oxidized, and necrotic lesions are observed in the leaves.^45^ This mechanism may be related to the stripping of cuticular waxes and rapid desiccation caused by cell leakage after membrane disruption.^46,47^ These effects represent the most established and consistently observed responses following PA application and form the basis for its classification as a contact herbicide.^48–50^

As a contact herbicide that induces rapid, nonspecific membrane disruption, PA does not rely on a single biochemical target and is therefore considered less prone to selecting for target-site resistance compared with systemic herbicides that act on specific enzymatic pathways. Consequently, there was no significant translocation in plants and no root uptake. López-Gonzáles et al. presented a timeline reporting the mechanism of action of PA. Lederer et al. proposed that PA can be intercalated in the lipid bilayer, inducing membranotropic destabilization (light-independent) and membrane peroxidation from photosensitized chlorophyll (light-dependent). However, Lebecque et al. concluded that PA does not have an alkyl chain long enough to destabilize membrane lipids.

Taken together, these studies support membrane disruption and rapid desiccation as the primary and immediate effects of PA, although the precise molecular interactions at the membrane level remain under debate. Beyond these established phytotoxic effects characteristic of contact herbicides, more recent studies have suggested that additional physiological processes may contribute to PA-induced phytotoxicity under controlled experimental conditions.

### 3.2 Emerging Hypotheses Involving Secondary Physiological Processes

López-Gonzáles et al. observed that azaleic acid, a dicarboxylic acid, can interfere with auxin transport by acting as an aliphatic carboxylic acid, similar to carboxylic acid. López-Gonzáles et al. conducted a similar investigation on PA. They found that the membrane could also be modulated by protein interactions rather than by lipids. These studies were conducted in Petri dishes containing *Arabidopsis thaliana* (L.) Heynh. Columbia (Col-0) plants were used as the plant model. This experiment was conducted under controlled conditions, ensuring high reproducibility. However, the observed effects may not fully translate to weed species, which often exhibit greater physiological variability and stress tolerance, making their responses more complex and less predictable. After plant exposure, increases in secondary and adventitious roots, torsion, gravitropism, and phytotoxicity were observed. These results were related to alterations in the ethylene balance and auxin transport mediated by the PIN1 protein, which indirectly reduced auxin in the shoot and increased its accumulation in the roots. Irregular and incomplete cell walls were observed, as with other auxin herbicides, and mitochondrial elongation was caused by ROS produced from auxin accumulation in the roots. Based on these observations, PA has been proposed to involve auxin-related signaling pathways, potentially contributing to its phytotoxic effects through a complex and indirect mechanism.

The proposed cellular and physiological effects in Figure 2. Auxin-mimicking herbicides have slower symptoms and effects on plants than contact herbicides. In the leaves, PA appears to cause rapid leaf damage. The inhibition of auxin transport is one of the different processes involved in a cascade of cell damage that leads to ROS production and membrane disruption. At the penetration level, no information is available for PA, and the mechanisms by which it crosses the cuticular barrier to reach the cell membranes and its site of action (unknown).

**Figure 2:**
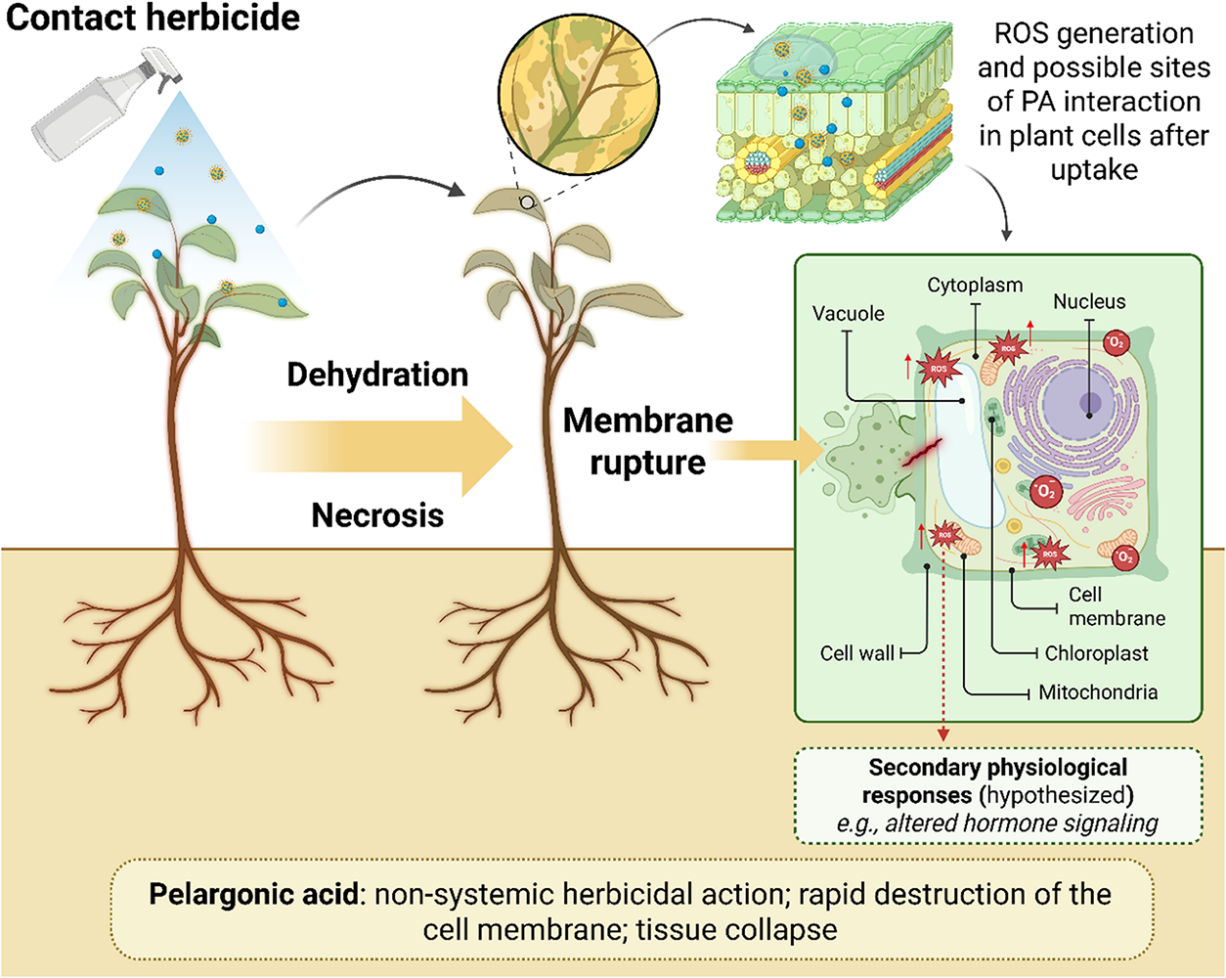
Schematic representation of the herbicidal action mechanisms of pelargonic acid (PA) after foliar spraying. Initially, PA is absorbed and penetrates the lipid bilayer of cell membranes, leading to leakage of cytoplasmic contents. This interaction triggers excessive generation of reactive oxygen species (ROS) in key organelles such as chloroplasts, mitochondria, and the vacuole. Oxidative stress, combined with loss of membrane integrity, leads to cell dehydration, tissue necrosis, and eventual plant collapse. Potential secondary physiological responses are shown schematically and should be interpreted as hypothesized downstream processes.

According to Coleman and Penner, hydrocarbon oils have been used as contact herbicides for more than half a century. Similarly, this type of herbicide action can be explained and extrapolated to the action of fatty acids, such as the PA natural molecule. Organic farmers use this herbicide as a landscape product (desiccant or defoliant).^51^ As a bioherbicide, PA (C9) can be more effective as a desiccant for beans than short-chain fatty acids and is comparable to the fast-acting paraquat and glufosinate.^62^ Emulsifiers and adjuvants can enhance fatty acid activity.

Glyphosate and PA are high-dose synergistic combinations that can replace paraquat.^52–55^ This dose-dependent effect has been observed in several studies. Pline et al. found that 3% (v/v) PA had no added efficacy in solutions containing glyphosate or glufosinate, which resulted in a reduction in soybean fresh weight. Kay reported that the SePRO Corp. tested PA as an adjuvant (SP1001) for glyphosate to increase its use in aquatic weed control. They evaluated the effects of up to 10% (v/v) PA alone and up to 3% PA mixed with glyphosate against cattail (*Typha latifolia* L.) and alligatorweed (*Alternanthera philoxeroides* (Mart.) Griseb.). When used alone, PA provided only transient suppression, with early plant injury followed by regrowth. When used in a mixture, it did not increase weed control, and its use as an adjuvant was not economically feasible.

PA was tested multiple times as a herbicide in a mixture, and its dose dependence was demonstrated. Wehtj et al. indicated that doses up to 1120 g acid equivalent (a.e) ha^–1^ were not effective, and their use was not justified. For *Phyllanthus tenellus* and *Cyperus esculentus*, a synergistic effect on early visual damage was observed, and the authors suggested that the selectivity of PA was related to the target. Compared with glyphosate (748 g i.a. active ingredient ha^–1^), pyraflufen (1085 g i.a. ha^–1^), and paraquat (2062 g i.a. ha^–1^), PA applied up to 5200 g i.a. ha^–1^ was not the best option for controlling the six weed species.^56,57^ Pyraflufen has been suggested as a potential substitute for paraquat derivatives in the preparation of fire-break tracer lines in South Africa.

After decades of discussion regarding the viability of PA use, commercial products have reached the market. In Europe, the emulsion of PA, Beloukha (Belchim Crop Protection—Technologielaan 7, 1840 Londerzeel, Belgium), gained prominence. In Italy, using the same formulation, Pannacci et al. reported different levels of susceptibility for 11 winter and summer weed species, with EC_50_ from 2600 to more than 21,800 g a.i. ha^–1^. The effectiveness of Beloukha has also been demonstrated by Kanatas et al. in controlling *Echinochloa crus-galli* (L.) P. Beauv. and *Sorghum halepense* (L.) Pers., at 7260 g a.i. ha^–1^, with 85–92% fresh weight biomass reduction. Adding adjuvant or microcapsules of caraway essential oil did not affect these results. Travlos et al. compared several PA herbicides, including Beloukha, Finalsan Ultima (W. Neudorff GmbH KG, Emmerthal, Germany), essential oils (mukuna, lemongrass, and pine oil), and mixtures of these. They found that natural products with high concentrations of PA were able to increase the control of grass weeds up to 10,000 g i.a. ha^–1^, and that broadleaf weeds were more susceptible, including at lower doses. Weed regrowth, which is common after PA application, can be avoided using the Finalsan Ultima formulation, which contains maleic hydrazide as a growth inhibitor. A synergistic effect was observed for mukuna oil and the elimination of almost all cleaver plants (*Galium aparine*).

Despite advances in understanding its physiological effects, PA has been identified in recent decades as a potential desiccant herbicide and is now reaching the market. Conventional organic agriculture is recommended as a sustainable weed management tool. As a substitute for paraquat and glyphosate in many regions, its commercialization is currently limited and can be further explored in terms of formulation, target plants, and mode of application.

In addition to its biological effects, the environmental fate of PA has been investigated. The European Food Safety Authority^58^ showed that the persistence of PA in the soil was low under aerobic conditions and in the absence of light. This suggests that microorganisms degrade the molecules through the sequential elimination of C_2_ fragments, that is, the main metabolites of PA in the soil are other shorter-chain fatty acids. The authors also highlight in additional studies that in an aquatic environment, the resulting DT50 value was 1.6 days, reinforcing the low persistence in the environment.

Poiger et al. demonstrated that the resulting DT50 value was less than 1 day, with even faster rates observed in agricultural soil (1.5 h). This indicates that this rate is relative to the concentration and characteristics of the soil. PA adsorption depends on pH, as expected for a weak acid (p*K*_a_, 4.94–5.0), with more intense adsorption at lower pH values. Restu et al. investigated the destruction of PAs in soil. The authors found that the elimination remaining after 3 days of treatment was 156.1 ppm, and the value was reduced in 7 days to 83.93 ppm. This suggests low environmental persistence and potential biodegradation, reinforcing its suitability for organic farming and repeated applications without long-term soil contamination.

Soil properties, such as organic matter content, which interferes with herbicide mobility, mineral composition, cation exchange capacity (CEC), and pH, influence herbicide retention dynamics, the main process that determines soil behavior.^61^ This degradation is primarily mediated by microbial activity, and its interaction with organic matter can further influence its bioavailability and degradation rate.

PA does not exhibit residual herbicidal activity in the soil, as its initial mode of action is based on contact, rather than systemic absorption or inhibition of root functions. From an environmental perspective, its rapid degradation, low toxicity, and low accumulation in the environment make it a promising alternative for sustainable weed management. Photodegradation and hydrolysis are also relevant under field conditions, since PA has a reported half-life of approximately 1–2 d in aqueous systems and degrades faster when exposed to sunlight, reinforcing its classification as a biodegradable and low-risk compound.^58,59^

Despite increasing efforts to elucidate its herbicidal mode of action, the mode of action of pelargonic acid remains only partially resolved. Current evidence robustly supports its primary contact activity, driven by membrane disruption, cellular leakage, and rapid tissue desiccation, which helps explain its fast burndown effect under field conditions. However, much of the mechanistic literature relies on simplified experimental systems and model species, with limited validation in agronomically relevant weeds. Accordingly, essential uncertainties remain regarding the molecular targets of pelargonic acid, its penetration across the cuticular barrier, and the relevance of secondary processes, such as auxin interference, under practical use conditions. To move beyond these limitations, future studies should prioritize weed species rather than model plants and combine physiological observations with biochemical indicators of oxidative stress and imaging-based analyses performed under application scenarios closer to field reality. This level of experimental detail is necessary to strengthen mechanistic interpretations and to link greenhouse findings with field-level performance better.

## 4 Engineered Delivery Systems for PA

### 4.1 Conceptual Background and Formulation Challenges

Innovative strategies are being developed to meet the demands of sustainable agriculture and promote the use of less hazardous agricultural inputs and environmentally friendly compounds. Among these are precision agriculture, genetic modifications through gene editing, and biobased approaches.^62^ Nanotechnology is an upcoming tool for sustainable agriculture, offering solutions to address the challenges affecting agricultural issues. The innovative properties of nanoparticles, such as their small size, increased surface-area-to-volume ratio, superficial quantum effects, enhanced reactivity, and tunable physical, electrical, magnetic, and optical properties, make them highly valuable for agricultural applications.^63,64^

Nanoparticles have been widely explored in agriculture for various purposes, including their roles in nanobiosensors, plant growth enhancement, nanofertilizers, nanopesticides, nutrient management, and phytopathogen protection.^65,66^

Nanotechnology is being investigated to address key agricultural issues, including efficient water management, removal of harmful substances, nutrient delivery, and contaminant detection. The aim is to mitigate health risks and reduce costs associated with agricultural chemical practices.^67^ Their physicochemical properties, combined with advantages such as extended shelf life and higher efficiency owing to targeted delivery at lower dosages, make them preferable alternatives to traditional methods. By incorporating sustainable materials such as lipids, natural polymers, and polysaccharides, green nanotechnology is advancing the development of innovative and eco-friendly nanoformulations, particularly for nanobased pesticides and plant disease management.^19,68–71^

Natural compounds play a crucial role in the development of pharmaceuticals and crop protection agents. Over the last 30 years, it has been estimated that natural compounds have contributed to approximately 35% of drugs newly approved by the FDA.^72^ Natural compounds, along with their synthetic derivatives and nature-inspired compounds, account for approximately 17% of all crop protection agents.^73–75^ Several natural compounds that are widely used in agriculture originate from plant and microbial metabolites, either as extracts or as isolated compounds. These compounds include lactones, pyrethrins, tripeptides, phenolic and polyphenolic compounds, alcohols, essential oils from various plant sources, and active terpenes. Among these, PA, a naturally occurring nine-carbon fatty acid, has gained attention as a bioherbicide because of its ability to rapidly desiccate plant tissues.^19,60^ Despite its effectiveness, the broader application of PA, like many other natural compounds, is limited by factors such as its high volatility, low water solubility, thermal instability, and nontargeted delivery.^9,19,60^

Nanotechnology is a promising approach to enhance the effectiveness of natural compounds by improving their stability, increasing treatment efficiency, reducing the required dosages, and enabling sustained and/or controlled release through encapsulation in several matrices. Together, these considerations highlight the importance of engineered delivery systems to overcome intrinsic formulation challenges of pelargonic acid and support its integration into sustainable weed management strategies.

### 4.2 Carriers for PA: Synthesis and Characterization

The encapsulation of PA in micro- and nanocarriers is a promising strategy for enhancing physicochemical stability, bioavailability, and controlled release for agricultural applications. PA is widely recognized as a contact burndown herbicide that disrupts plant cell membranes and leads to rapid desiccation of green tissues, without a defined molecular target. However, their direct application faces challenges such as high volatility, water immiscibility, and rapid environmental degradation, which limit their long-term efficacy in the field.^19^ Several carriers, including polymeric nanoparticles, lipid-based systems, and biopolymeric matrices, offer sustainable approaches to overcome these limitations by protecting PA from premature degradation, reducing leaching, and improving targeted delivery.

Different studies have been conducted on the encapsulation of PA. White et al. (2021) evaluated pelargonic acid emulsions as postharvest sanitizers to reduce *Salmonella enterica* contamination on tomatoes. Emulsions formulated with Quillaja saponin (0.1% w/v) at 30–50 mM achieved >6 log CFU/g reduction after 7 days at 8 °C, outperforming chlorine and peroxyacetic acid. However, the treatment negatively affected fruit firmness, highlighting formulation challenges. Although not designed for weed control, these studies demonstrate the broader potential of PA delivery systems and provide insights into stability and application constraints relevant to agricultural formulations.

Restu et al. developed a lignin-based delivery system for PA to enhance its stability and herbicidal efficacy while reducing its environmental impact. The optimized PA-lignin emulsion, formulated at pH 5 with stirring at 5000 rpm, yielded a stable droplet size of 104.4 ± 0.9 nm and a zeta potential of −28.5 ± 1.3 mV, exhibiting no phase separation after 14 days. The formulations eliminated *Pennisetum purpureum* weeds within 1 day of application, with no regrowth observed after 7 d. Lignin functions as a biodegradable carrier, minimizing PA leaching and environmental contamination while degrading it into humic acid, which can improve soil quality. Residue analysis showed a decline in the PA concentration from 156.1 ppm at 3 day postapplication to 83.93 ppm after 7 days, confirming its degradability.

Pelargonic acid ester derivatives have been evaluated as sustainable alternatives, with emphasis on esters with methylated poly­(ethylene glycol) containing six ethylene oxide units (mPEG-6EO-PA). This compound exhibited phytotoxicity equal to or greater than that of PA in its free form, even at lower spray volumes, indicating its potential as an efficient herbicide molecule and its compatibility with low-impact environmental formulations.^76^

Despite recent advances, nanoformulations of natural herbicidal compounds, particularly PA, remain scarce. Most research focuses on synthetic molecules, leaving a substantial gap in the development of nanocarriers for natural bioactive compounds. This underexplored field presents both challenges and opportunities for innovation, particularly considering the urgent need for environmentally safe and efficient solutions in agriculture, especially for weed control. Studies involving the nanoencapsulation of PA and other natural compounds are important to overcome current formulation barriers and enhance their application in agriculture.

Research on engineered delivery systems has demonstrated many advantages, particularly in improving physicochemical stability, reducing volatility, and enhancing foliar retention. These advances represent an essential step toward overcoming the intrinsic limitations of free pelargonic acid and align well with sustainability-driven formulation strategies. Nevertheless, the current body of work remains fragmented and limited in scope. Most studies emphasize formulation stability or short-term efficacy, while systematic evaluations of absorption, translocation, release kinetics, and long-term weed control are still scarce. In addition, comparisons across carrier types are hindered by inconsistent experimental designs, application rates, and target species. To advance the field, future studies should adopt standardized evaluation frameworks that integrate formulation performance with plant physiological responses and environmental fate. Such an approach would enable meaningful cross-study comparisons and accelerate the translation of nanoenabled pelargonic acid formulations from laboratory concepts to practical agricultural solutions.

## 5 Patent Landscape of PA: An Overview

The patent landscape provides insights into technological development, innovation trends, and commercial interest surrounding pelargonic acid (PA) in crop protection. Patents were surveyed across major databases (Google Patents, Espacenet, and WIPO), focusing on documents published over the last 10 years and restricted to agricultural applications (Table S1).

Overall, the analysis of patented technologies reveals that most innovations involving pelargonic acid as a herbicide are concentrated at the formulation and use-combination levels rather than on new active molecules or novel modes of action. Core claims predominantly describe emulsifiable concentrates, surfactant-stabilized emulsions, low-viscosity aqueous systems, or synergistic combinations with other herbicides, to improve foliar coverage, persistence, and weed-control efficacy. This pattern reflects an effort to overcome the intrinsic limitations of pelargonic acid as a contact herbicide, particularly its low persistence and limited translocation.

A substantial group of patents focuses on formulation engineering to stabilize pelargonic acid in aqueous systems and improve its delivery on plant surfaces. Examples include JP2016190832A, which proposes low-viscosity aqueous emulsions using surfactants and inorganic or organic salts, and CN111406748A, which discloses plant-derived pelargonic acid formulations stabilized with poly­(oxyethylene ether) surfactants. Similarly, EP4025049A1 and KR102453553B1 describe concentrated emulsifiable compositions containing defined ratios of anionic and nonionic surfactants, solvents, and adjuvants, resulting in stable aqueous emulsions and enhanced desiccant activity under field conditions. Together, these patents illustrate a consistent trend toward improving the agronomic performance of pelargonic acid through formulation optimization rather than through molecular innovation.

Beyond formulation stability, several patents emphasize synergistic or combinatorial strategies to enhance herbicidal efficacy. IN202217042202 exemplifies this approach by claiming pelargonic acid–based herbicide compositions formulated with optimized coformulants to improve application stability and field performance. More explicitly, US20240365781A1 describes synergistic herbicidal compositions that combine pelargonic acid with the ALS inhibitor flazasulfuron, demonstrating improved weed control relative to the individual components. Likewise, WO2024165498A1 claims formulations combining pelargonic acid with other herbicidal actives or coformulants to broaden the weed control spectrum and enhance overall efficacy.

In line with this combinatorial trend, US20240415120A1 discloses a bioherbicide comprising pelargonic acid and a carbonyl compound, aiming to improve herbicidal efficacy while reducing environmental and health risks. These patents collectively indicate a growing interest in pairing pelargonic acid with complementary active ingredients to compensate for the limitations of contact herbicides, rather than altering the fundamental mode of action of PA.

Even when nanoparticles are not explicitly mentioned, applicants’ innovations converge to improve the stability and efficiency of the molecule, primarily through advances in formulation strategies (including emulsions, surfactant-stabilized mixtures, and synergistic blends). The annual distribution of patents (Figure 3) shows a steady increase in the number of filings over the past decade, with a clear peak in 2023. Herbicidal uses remain the dominant focus, followed by a smaller number of patents related to insecticidal, fungicidal, and other agricultural applications.

**Figure 3:**
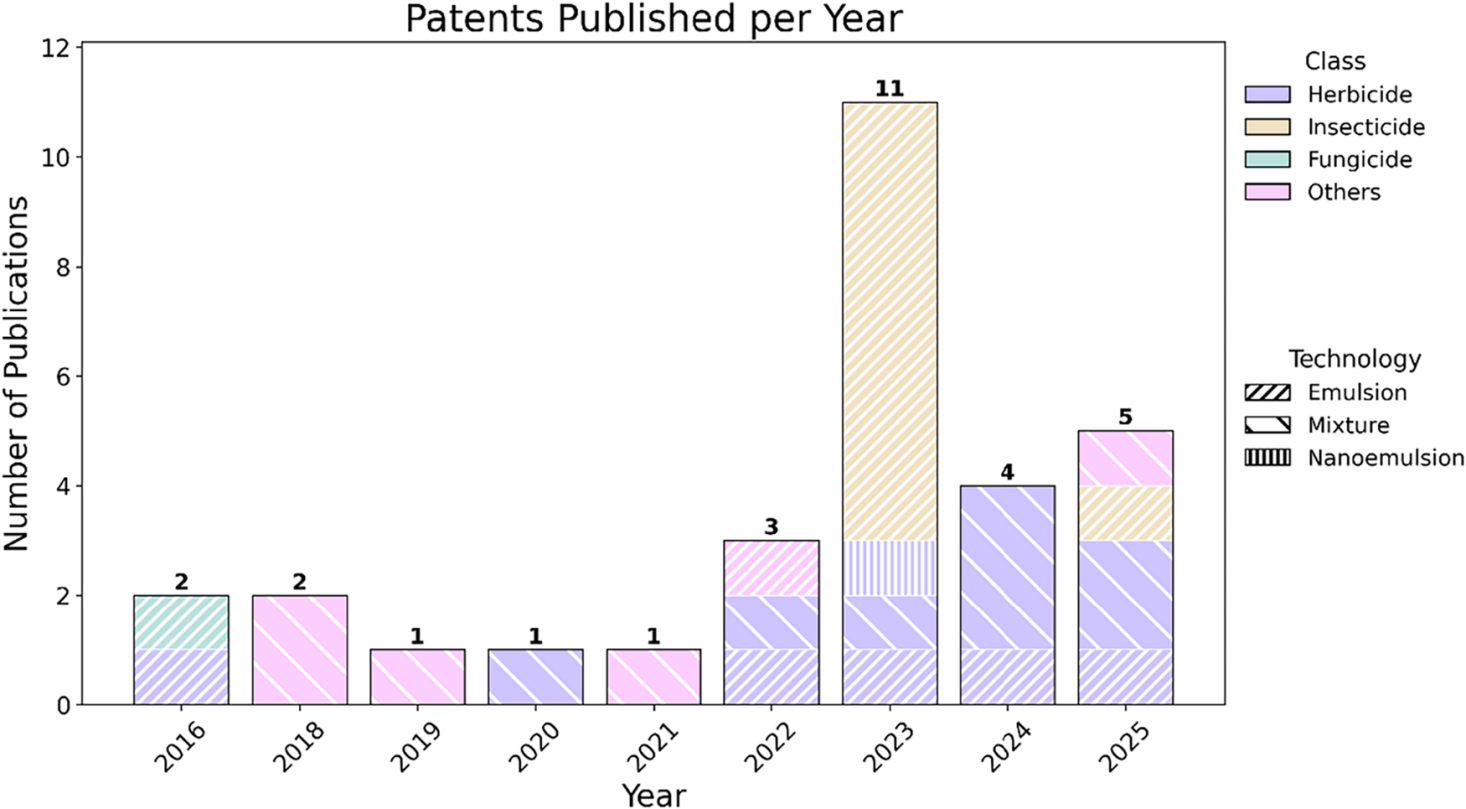
Annual distribution of patents involving pelargonic acid, covering documents published between 2016 and 2025 and retrieved from Google Patents, Espacenet, and WIPO databases.

This reflects a broader diversification of claimed technologies, as evidenced by the analysis of the patent descriptions summarized in Supporting Information (Table S1). This trend suggests a growing interest in adapting technologies based on pelargonic acid to different agrochemical contexts, likely driven by regulatory pressure on conventional synthetic pesticides and the increased demand for biobased alternatives, rather than a fundamental shift in the underlying mode of action.

Overall, the patent landscape indicates growing industrial interest in formulations based on pelargonic acid, particularly for herbicidal uses, reflecting the commercial relevance of this molecule. Despite this expansion, a substantial portion of the patents relies on incremental formulation adjustments, often centered on emulsification strategies or compositional changes, with limited mechanistic insight or validation under realistic agronomic conditions. This disconnect between patent claims and field-scale evidence highlights the need for more rigorous experimental support to strengthen technological robustness and practical applicability. Bridging this gap will be essential to ensure that patented technologies translate into effective, reliable, and scalable agricultural solutions.

## 6 Conclusions and Future Perspectives

The literature review conducted in this study demonstrates that PA is consolidating itself as a viable and promising alternative in the global scenario of plant management, driven by the growing demand for more sustainable solutions and the urgency to mitigate agricultural challenges. Its natural origin, rapid degradation in the environment, low toxicity, and the absence of residues in the soil make it a low-risk tool for the environment.

Despite its positive effects, the mode of action remains poorly understood. In general, PA acts as a contact herbicide, causing rapid dehydration, rupture of plant cell membranes, and physiological imbalances, providing an effective and fast-acting alternative. However, questions regarding the entry route, primary site of action, initial damage, and regulatory pathways remain unanswered. Some studies have altered ethylene balance and auxin transport via PIN1 protein and complex interactions, including a possible auxin-mimetic effect. Understanding how a molecule works is crucial for effective management and informed decision-making in the agricultural sector.

The transition to a more widespread use of PA is still limited by problems related to its physicochemical properties, such as high volatility and low water solubility, compared with conventional herbicides. In this context, all reported positive effects on plant control have been observed in smaller plants, and the impact on plants at different stages of development is unknown and requires in-depth investigation. The search for more efficient and sustainable synthetic routes, particularly those based on renewable sources such as oleic acid, is crucial to reducing dependence on petrochemical methods and to enhancing PA production more sustainably. In response to these challenges, nanotechnology is emerging as a fundamental alternative for next-generation PA formulations, particularly when combined with sustainable production strategies aligned with green chemistry principles, thereby enabling advanced and efficient delivery systems.

The encapsulation of PA in nanocarriers such as polymeric nanoparticles, lipid systems, and various matrices represents an effective strategy. These systems provide adequate protection for the active ingredient against degradation, acting as a protective barrier while increasing leaf adhesion, minimizing leaching, and enabling controlled and targeted release. Although studies on natural molecules such as PA are scarce, existing evidence suggests that nanotechnology will enable formulations with greater stability, enhanced efficacy, reduced application frequency, and lower environmental impact. Therefore, the prospects of using PA in agriculture are promising, although large-scale adoption will ultimately depend on economic feasibility and production scalability, particularly in low-margin agricultural systems. However, they require continuous research and development on several fronts, such as (i) methods for producing PA from renewable sources, seeking more efficient and economically viable processes; (ii) integration into integrated management strategies, exploring their use in rotation with other herbicides, in synergistic molecules, or in conjunction with cultural and biological practices, to optimize control and prevent resistance; (iii) regulation, as nanoformulations are developed, with clear and objective regulatory guidelines; and (iv) acceptance, highlighting the benefits of productivity, the environment, and the safety of these products. In this context, future studies integrating formulation performance, application frequency, and environmental behavior will be essential to benchmark PA-based technologies against conventional synthetic herbicides fully. These efforts should increasingly focus on field trials conducted across different seasons and locations, allowing direct comparisons between nanoparticles with PA, conventional PA products, and widely used synthetic herbicides under realistic agronomic conditions. In addition to weed control efficacy, parameters such as regrowth, application frequency, costs, and environmental behavior should be considered to support the practical evaluation of these systems. PA is a key component of sustainable plant management strategies envisioned in the near future.

## Supplementary Material



## Notes

Comprehensive compilation of pelargonic acid-related patents published between 2016 and 2025, including patent numbers, publication year, technological approaches, and reported agricultural applications (Table S1); this supporting material supports the patent landscape analysis presented in the manuscript by providing detailed information on formulation strategies and technological developments involving pelargonic acid (PDF)
