# Supplementary material for: Pelargonic Acid: From Historical Uses to Future Perspectives in Sustainable Agriculture
Source: J Agric Food Chem. 2026 Mar 3;74(12):9903–15. doi: 10.1021/acs.jafc.5c14438 (PMC13047689; doi:10.1021/acs.jafc.5c14438)
Supplement: Supplementary file 1 [file jf5c14438_si_001.pdf]

Pelargonic Acid: From Historical Uses to Future Perspectives in Sustainable Agriculture

Ana Cristina Preisler[1], Estefânia Vangelie Ramos Campos[1,2] , Vanessa Takeshita[1], Jéssica S. Rodrigues[1], Amanda de S. M. de Freitas[1], Brian Cintra Cardoso[1], Lais D. Battaglini[1], Leonardo Fernandes Fraceto[1*]

[1]Institute of Science and Technology, Sao Paulo State University, Av. Três de Março, 511 - Alto da Boa Vista, 18087-180, Sorocaba, SP, Brazil.

2 B. Nano Soluções Tecnológicas LTDA, Avenida Itavuvu 11777, Sorocaba, SP, Brazil.

SUPPLEMENTARY INFORMATION

Supplementary Table 1: Patents involving pelargonic acid (2016–2025) retrieved from major patent databases, detailing patent number, year, technology, and agricultural application. Herbicidal uses predominate, with additional insecticidal, fungicidal, algicidal, and other applications.

Patent	Year	Technology	Application
JP2016190832A	2016	Emulsion of Pelargonic Acid and Glyphosate	Broad spectrum weed control
KR101817513B1	2016	Formulation of Pelargonic Acid and clove essential oil	Fungus control
KR101812220000*	2018	Formulation of Pelargonic Acid and oxidizing agents	Marine algae
US11395488B2	2018	Formulation of Pelargonic Acid with non-ionic surfactants for control of unwanted sprouting	Sprouting control in ornamental and commercial plants
JP2019001777A	2019	Formulation of Pelargonic Acid combined with phenolic compounds	Moss removal
WO2019030062	2019	Formulation of Saturated Aliphatic Monocarboxylic Acids comprising Pelargonic Acid (20–99%) with Ketoacids	Weed control
CN111406748A	2020	Formulation of Pelargonic Acid and synergistic components	Broad spectrum weed control
EP4025049A1	2020	Emulsifiable composition comprising pelargonic acid	Weed control

CN108300463A	2021	Pelargonic Acid to introduce hydrotropic groups in the synthesis of graphene quantum dots	Applications in advanced materials
SK202250051	2022	Emulsion for concrete with Pelargonic Acid and vegetable oils	Moss and fungi on concrete surfaces
IN202217042202	2022	Formulation of Pelargonic Acid with anionic and non-ionic surfactants	Broad spectrum weed control and growth regulator
KR102453553B1	2022	Formulation of Pelargonic Acid and sodium alginate	Broad spectrum weed control and repellent action
CN117044734A	2023	Emulsion for slow release of Pelargonic Acid, Terbuthylazine and Caprylic Acid	Broad spectrum weed control
JP7249566B2	2023	Nanoemulsion of Pelargonic Acid and glyphosate	Broad spectrum weed control and insect repellency
JP2023032414A	2023	Formulation of Pelargonic Acid and glyphosate salts stabilized with cationic surfactants	Broad spectrum weed control
US2025134100A1	2023	Use of Pelargonic Acid as insecticide/acaricide	Control of insects and mites in perennial crops
US2025160320A1	2023	Use of Pelargonic Acid as insecticide/acaricide	Control of insects and mites in corn and sorghum crops
US2025127163A1	2023	Use of Pelargonic Acid as insecticide/acaricide	Control of insects and mites in soybean crops
US2025127164A1	2023	Use of Pelargonic Acid as insecticide/acaricide	Control of insects and mites in cotton crops
WO2023148033A1	2023	Use of Pelargonic Acid as insecticide/acaricide	Control of insects and mites in canola crops
US2025127162A1	2023	Use of Pelargonic Acid as insecticide/acaricide	Control of insects and mites in rice crops
US2025160322A1	2023	Use of Pelargonic Acid as insecticide/acaricide	Control of insects and mites in cereals

US2025143299A1	2023	Use of Pelargonic Acid as insecticide/acaricide	Control of insects and mites in horticultural crops
US2024365781A1	2024	Formulation of Pelargonic Acid and Flazasulfuron	Broad spectrum weed control
WO2024165498A1	2024	Formulation of Pelargonic Acid with PPO and PSII inhibitors	Broad spectrum weed control
US2024415120A1	2024	Synergism of Pelargonic Acid with unsaturated carbonic compounds	Broad spectrum weed control
US2023354816A1	2024	Synergistic composition of colina salt of Pelargonic Acid with elemental sulfur	Broad spectrum weed control and fungi
US2025072420A1	2025	Formulation of Pelargonic Acid and synergistic herbicides	Dicotyledonous weeds
NZ821408	2025	Formulation of Pelargonic Acid, polar solvent and aqueous vehicle	Weeds, algae and fungi
US2025127171A1	2025	Formulation of Pelargonic Acid and L-glufosinate	Broad spectrum weed control
EP4501112A1	2025	Use of Pelargonic Acid for natural plant defense against biotic and abiotic stresses	Plant defense system against biotic and abiotic stresses
WO2025026815A1	2025	Use of Pelargonic Acid as insecticide/acaricide	Control of insects and mites in various crops
